# Changes in Psychological Burden and Quality of Life During In Vivo Diagnostic Evaluation for Immediate Drug Hypersensitivity: A Prospective Stepwise Study

**DOI:** 10.3390/jcm15207964

**Published:** 2026-10-15

**Authors:** Ayşe Feyza Aslan, Semra Demir, Pelin Korkmaz, İlkim Deniz Toprak, Nevzat Kahveci, Merve Hörmet İğde, Mehmet Emin Sezgin, Bircan Erden, Deniz Eyice Karabacak, Şule Çelik Kamacı, Andaç Karadeniz, Yavuz Erkam Kesgin, Selin Ece Güzel, Merve Dilşad Atasever, Leyla Bölek, Esra Kaya, Osman Ozan Yeğit, Derya Ünal, Aslı Gelincik

**Affiliations:** Division of Immunology and Allergic Diseases, Department of Internal Medicine, Istanbul Faculty of Medicine, Istanbul University, Istanbul 34093, Türkiye

**Keywords:** drug hypersensitivity, drug provocation test, quality of life, drug hypersensitivity quality of life questionnaire, anxiety

## Abstract

**Background/Objective:** The psychological impact of in vivo diagnostic procedures remains poorly understood. We assessed changes in psychological distress and drug hypersensitivity-related quality of life (QoL) throughout a diagnostic evaluation for suspected immediate drug hypersensitivity reactions (IDHRs) and aimed to identify predictors of persistent psychological burden. **Methods:** This single-center prospective observational longitudinal study included 100 consecutive adults undergoing evaluation for suspected IDHRs. Psychological distress and QoL were assessed using the Depression Anxiety Stress Scale-21 (DASS-21) and the Drug Hypersensitivity Quality of Life Questionnaire (DrHy-Q). Assessments were performed before and after each diagnostic phase, including skin tests (STs), placebo, and drug provocation tests (DPTs). **Results:** The mean age was 44.2 ± 12.5 years, and women comprised 72% of the cohort. Depression, anxiety, stress, and DrHy-Q scores significantly decreased after ST, placebo, and DPT compared with baseline measurements. Across diagnostic stages, depression, anxiety, stress, and DrHy-Q scores were significantly lower during the DPT phase than during the ST and placebo phase in both pre-test and post-test measurements (*p* < 0.01 for pairwise comparisons). The number of prior HSRs showed a weak but significant positive correlation with DrHy-Q scores. In multivariable linear regression analysis, the number of prior HSRs was the only independent predictor of post-DPT DrHy-Q scores (β = 3.43, 95% CI 1.36–5.50, *p* = 0.001). Patients who avoided the provoked drug despite a negative DPT had higher DPT-post depression, anxiety, stress, and DrHy-Q scores. **Conclusions:** Psychological burden progressively decreased over the course of the stepwise diagnostic evaluation for suspected IDHRs, although patients with repeated reactions may benefit from additional support.

## 1. Introduction

Drug hypersensitivity reactions (DHRs) are a common concern in clinical practice and a major cause of morbidity, mortality, and healthcare costs. Although patients often describe their adverse experiences as “drug allergies,” only a small portion of reported reactions are true immune-mediated hypersensitivities. Mislabeling adverse drug reactions (ADRs) as allergies results in unnecessary drug avoidance, overuse of broad-spectrum or expensive alternatives, and a higher risk of antimicrobial resistance [1,2]. Epidemiological studies show ADRs in about 10–20% of hospitalized patients, yet only a minority of reported drug allergies are ultimately confirmed [1,2,3,4].

Many patients report reactions to multiple different drug classes, giving rise to classifications such as multiple drug intolerance syndrome (MDIS), defined by non-immunologic intolerance to three or more drug classes, and multiple drug allergy syndrome (MDAS), characterized by hypersensitivity reactions (HSRs) to at least two classes [5,6,7,8]. Although uncommon, these patterns most frequently involve antibiotics and nonsteroidal anti-inflammatory drugs (NSAIDs) [9]. Psychological burden is increasingly recognized in patients with DHRs, with studies demonstrating increased anxiety and depression and significant impairment in health-related QoL, particularly among patients with severe reactions or drug-induced anaphylaxis [10,11,12].

Beyond physical symptoms, suspected drug hypersensitivity can impose a substantial emotional and functional burden and adversely affect health-related QoL. The Drug Hypersensitivity Quality of Life Questionnaire (DrHy-Q) was specifically developed to assess this burden, and recent prospective evidence has further supported its validity as a patient-reported measure of health-related QoL in patients with suspected or confirmed DHRs [13,14,15,16]. More recently, a large international multicenter study demonstrated impaired DrHy-Q among patients carrying a penicillin allergy label, with greater impairment observed in women and those with additional antimicrobial allergy labels [17]. However, little is known about how psychological distress and drug-related QoL change throughout the stepwise diagnostic process. Skin tests (STs), placebo exposure, and drug provocation tests (DPTs) are essential for confirming or excluding DHRs, yet these procedures themselves may heighten anxiety due to fear of reaction [18,19].

Despite the centrality of these diagnostic steps in allergy practice, no previous study has systematically evaluated how psychological measures fluctuate across phases of testing or which patient characteristics predict greater psychological burden. Therefore, this study aimed to assess changes in psychological distress and the DrHy-Q through a stepwise evaluation across each stage of the diagnostic work-up, including ST, placebo administration, and DPT, and to identify clinical predictors associated with increased psychological burden among patients with suspected DHRs.

## 2. Materials and Methods

### 2.1. Study Design and Study Population

This single-center, prospective observational study was conducted at the Division of Adult Immunology and Allergic Diseases of Istanbul Faculty of Medicine between 15 November 2025 and 10 January 2026. The study population consisted of 100 consecutive adult patients (≥18 years) with a history of IDHRs and an indication for diagnostic testing who presented to the study center during the inclusion period.

All 100 patients underwent diagnostic procedures, including ST, placebo administration, and DPT. Eligible patients were required to have a clinical history compatible with an IDHR and to be able to complete the Turkish versions of the DrHy-Q and Depression Anxiety Stress Scale-21 (DASS-21) questionnaires [15,20]. Exclusion criteria included age < 18 years, non-immediate hypersensitivity reactions (NIHRs), inability to provide consent or complete questionnaires, significant psychiatric comorbidity that could interfere with psychological assessment, pregnancy or lactation, immunosuppressive therapy, or uncontrolled systemic disease precluding diagnostic testing.

Clinical data were collected using a standardized case report form. Variables included demographic characteristics (age, sex, education level, residence), detailed DHR history, clinical manifestations, comorbid conditions, laboratory findings, and diagnostic test results. For subgroup analyses, age was categorized into three groups as 18–39, 40–59, and ≥60 years to reflect young adulthood, midlife, and older adulthood, consistent with commonly used age strata in clinical research [21].

Occupational categories were grouped according to the nature of work and working conditions involving white-collar, blue-collar, healthcare workers, and homemakers to enhance interpretability. The reaction history included the number, timing, and characteristics of prior IDHRs and the suspected drugs and drug classes, as well as the route and latency of each reaction. Clinical features such as involved organ systems, reaction severity, and the presence of anaphylaxis were documented. Systemic reaction severity was graded using the World Allergy Organization (WAO) anaphylaxis grading system. Reactions classified as grade 3 or higher were considered anaphylaxis [22].

Comorbid conditions, including chronic medical diseases, atopic status, and coexisting allergic disorders, were recorded. Atopy was defined as sensitization to at least one common aeroallergen detected by skin prick testing or specific IgE [23].

### 2.2. Diagnostic Procedures

All diagnostic procedures were performed according to routine clinical practice and current national and international drug allergy guidelines [18,24,25]. STs, placebo administration, and DPTs were conducted on separate days, with at least one week in between to ensure independent assessment of depression, anxiety, stress, and the DrHy-Q at each phase. STs with culprit and alternative drugs were performed using available drug formulations (oral or parenteral) at previously established non-irritating concentrations, in accordance with published recommendations [24,25].

DPTs were performed in a monitored hospital setting using a single-blind protocol. Placebo administration was carried out using the same protocol and clinical setting as DPT. Patients were unaware of whether the placebo or the active drug was being administered, whereas the clinical team was aware of the administered substance. During DPT, the test drug, either the suspected culprit drug or a clinically appropriate alternative, was administered in incrementally increasing doses. In patients with non-severe immediate reactions limited to skin symptoms, the starting dose did not exceed 1/10 of the target single therapeutic dose, whereas in patients with a history of anaphylaxis, the starting dose did not exceed 1/100 of the target dose. Subsequent doses were administered at intervals of at least 30 min until the target therapeutic dose was reached, with patients observed throughout the procedure and for at least six hours afterward. A DPT was considered positive when objective signs and symptoms compatible with the index hypersensitivity reaction occurred; isolated subjective symptoms without accompanying objective findings were not considered sufficient to define a positive DPT. A DPT was considered negative when no hypersensitivity reaction developed [18].

### 2.3. Assessment of Psychological Status and Quality of Life

Psychological status and drug-related QoL were assessed using the DASS-21 and the validated Turkish versions of the DrHy-Q, respectively. The DrHy-Q measures the impact of suspected DHRs on emotional and functional well-being, with higher scores indicating poorer QoL [15]. The DASS-21 evaluates depression, anxiety, and stress using a four-point Likert scale comprising three subscales, each consisting of seven items [20]. The two instruments were used as complementary measures: the DASS-21 assesses general psychological burden, whereas the DrHy-Q evaluates the drug hypersensitivity-specific impact on QoL. Their combined use therefore allowed assessment of both general psychological symptoms and disease-specific quality-of-life impairment during the diagnostic process.

Both instruments were administered to all patients before and after each diagnostic step, including STs, placebo administration, and DPTs, to assess phase-specific changes in disease burden. All questionnaires were self-administered and did not require psychiatric supervision [15,20]. After completing the diagnostic work-up, patients were contacted to determine whether they later used the drug deemed safe during DPTs, and reasons for avoidance were recorded when applicable.

### 2.4. Statistical Analysis

Statistical analyses were performed using IBM SPSS Statistics version 25.0 (IBM Corp., Armonk, NY, USA). Continuous variables are reported as mean ± standard deviation (SD) or median (interquartile range, IQR), depending on distribution, and categorical variables as frequencies and percentages. Normality was assessed using the Shapiro–Wilk test. Given the exploratory nature of the study, no formal a priori sample-size calculation was performed.

Within-subject comparisons of psychological scores across diagnostic phases were performed using nonparametric tests (Friedman test with post hoc Wilcoxon signed-rank tests), as appropriate. Post hoc pairwise comparisons were adjusted using the Bonferroni method. Between-group comparisons of psychological outcome scores were performed using the Mann–Whitney U test for two independent groups and the Kruskal–Wallis test for three independent groups, as appropriate. Associations between psychological measures and clinical variables were evaluated using Spearman correlation. Correlation coefficients were interpreted as weak (ρ = 0.20–0.39), moderate (ρ = 0.40–0.59), or strong (ρ ≥ 0.60), in accordance with commonly accepted conventions.

To additionally account for the longitudinal within-patient structure, linear mixed-effects models were fitted separately for depression, anxiety, stress, and DrHy-Q scores, with assessment time, sex, age group, and number of prior HSRs entered as fixed effects and a subject-specific random intercept. Models were estimated using restricted maximum likelihood with the Satterthwaite approximation for degrees of freedom, and Bonferroni adjustment was applied to pairwise comparisons of estimated marginal means.

Multivariable linear regression analyses were performed to identify independent predictors of DPT-post depression, anxiety, stress, and DrHy-Q scores. Based on clinical relevance and findings from univariate analyses, age group, sex, and number of prior HSRs were included as covariates. Given the sample size, the models were restricted to these clinically relevant covariates to reduce the risk of overfitting. Multicollinearity and influential observations were assessed using variance inflation factors (VIFs) and Cook’s distance, respectively. In addition, 2000-sample bias-corrected and accelerated (BCa) bootstrapping was performed as a sensitivity analysis to assess the stability of the regression estimates. All tests were two-tailed, and statistical significance was defined as *p* < 0.05.

## 3. Results

The mean age of the patients was 44.2 ± 12.5 years, and the majority were women (72% of the cohort). Atopy was identified in 22% of patients, most commonly due to house dust mite sensitization (14%). Regarding occupational status, 46% of patients were homemakers, while 28% were employed in white-collar occupations. Comorbidities were present in 44% of participants, with hypertension, diabetes mellitus, and asthma being the most frequently reported conditions. Detailed demographic and clinical characteristics are summarized in Table 1.

As illustrated in Figure 1, pruritus, flushing, and urticaria were the most frequently observed manifestations. Less frequent manifestations included hypotension, neurologic findings, emesis, vomiting, chest pain, and cardiopulmonary arrest.

In this cohort, 51% of patients reported a prior history of anaphylaxis, and the most frequent reaction severity was grade 3, observed in 46% of cases. The interval between the index HSR and clinical evaluation showed substantial heterogeneity, with a median of 24 months, whereas the time since the most recent reaction was markedly shorter (median 6 months), as shown in Table 2. A substantial proportion of patients experienced more than one HSR, and many reacted to multiple drug classes. Regarding symptom onset, 59% of reactions occurred within 60 min after drug exposure and 41% within 1–6 h. Oral administration was the predominant route (85%), while intravenous (18%), intramuscular (3%), subcutaneous (1%), and topical (4%) exposures were less frequent. The most common suspected culprit drugs were β-lactam antibiotics (44%) and NSAIDs (42%), followed by non-β-lactam antibiotics (20%). A detailed summary of these clinical characteristics is provided in Table 2.

As shown in Figure 2 and Table 3, significant within-phase reductions were observed in all psychological outcomes and DrHy-Q scores after each diagnostic step. Depression scores showed a modest but significant reduction after ST (*p* = 0.032), followed by larger decreases after placebo (3.0 to 2.0, *p* < 0.001) and DPT (2.0 to 1.0, *p* = 0.002). Similar reductions were observed for anxiety (ST: *p* = 0.006; placebo: *p* < 0.001; DPT: *p* < 0.001), stress (ST: *p* = 0.007; placebo: *p* < 0.001; DPT: *p* < 0.001), and DrHy-Q scores (*p* < 0.001).

Across diagnostic stages, pre-test scores differed significantly for depression, anxiety, stress, and DrHy-Q (*p* < 0.001). Bonferroni-adjusted analyses showed that DPT-pre scores were consistently lower than both ST-pre and placebo-pre scores for all outcomes (adjusted *p* ≤ 0.003). Post-test comparisons also demonstrated significant differences across phases (*p* < 0.001), with DPT-post scores significantly lower than both ST-post and placebo-post scores for depression, anxiety, stress, and DrHy-Q (adjusted *p* ≤ 0.001). Detailed results are presented in Table 4.

The number of prior HSRs was not associated with pre-test depression, anxiety, or stress scores across the STs, placebo, or DPT phases. In contrast, a consistent weak positive correlation was observed between prior reaction number and DrHy-Q scores at all three time points (STs: ρ = 0.269, *p* = 0.007; placebo: ρ = 0.290, *p* = 0.003; DPT: ρ = 0.290, *p* = 0.003), indicating that patients with a higher number of previous reactions reported significantly greater impairment in DrHy-Q. Reaction number was not associated with post-test depression, anxiety, or stress scores at any diagnostic stage. In contrast, post-test DrHy-Q scores showed weak but statistically significant positive correlations with the number of prior reactions following STs (ρ = 0.249, *p* = 0.012), placebo (ρ = 0.245, *p* = 0.014), and DPT (ρ = 0.328, *p* = 0.001). These associations are illustrated in Figure 3 and detailed in Supplementary Table S1.

The HSR grade was not associated with depression or DrHy-Q scores at any stage of the diagnostic process. Weak inverse correlations were observed between HSR grade and anxiety at the ST-pre and placebo-pre stages (ρ = −0.236, *p* = 0.018 for both), as well as between HSR grade and stress at several stages, including ST-pre (ρ = −0.232, *p* = 0.021), ST-post (ρ = −0.254, *p* = 0.011), and DPT-pre (ρ = −0.253, *p* = 0.011); however, these associations were modest and inconsistent. Stress levels differed significantly across age groups at all stages of the diagnostic process (*p* range: 0.008–0.046), with patients aged 40–59 years consistently exhibiting the highest stress scores. At ST-pre, median (IQR) stress scores were 2.0 (0.0–5.0), 5.0 (2.0–8.0), and 2.0 (1.0–3.5) in patients aged 18–39, 40–59, and ≥60 years, respectively; corresponding DPT-post values were 1.0 (0.0–3.0), 3.0 (1.0–6.5), and 1.0 (1.0–4.0). No significant age-related differences were observed for depression, anxiety, or DrHy-Q scores. Detailed results are provided in Supplementary Table S1, and age-related differences in stress scores across the diagnostic stages are illustrated in Figure 4.

Female patients had significantly higher depression, anxiety, and DrHy-Q scores at ST-pre (*p* = 0.042, *p* = 0.020, and *p* = 0.012, respectively) and placebo-pre time points (*p* = 0.031, *p* = 0.005, and *p* = 0.002, respectively), with stress scores showing a non-significant trend toward higher values. DPT-pre sex differences were observed only for DrHy-Q, which remained higher in women (*p* = 0.026).

Post-test assessments showed a similar pattern: Females reported higher anxiety and DrHy-Q ST-post (*p* = 0.01 and *p* = 0.003, respectively) and higher depression, anxiety, and DrHy-Q placebo-post (*p* = 0.022, *p* = 0.019, and *p* = 0.003, respectively), whereas stress remained non-significant. DPT-post sex differences were no longer evident for depression, anxiety, or stress, although DrHy-Q remained higher among women (*p* = 0.024). Detailed results are summarized in Supplementary Table S2.

The interval since the last hypersensitivity reaction (0–6, 6–12, or >12 months) was not associated with any psychological measure at any phase of testing (*p* > 0.05). Reaction-onset time, categorized as 0–1 h and 1–6 h, was not associated with depression, anxiety, stress, or DrHy-Q scores at any diagnostic stage (all *p* > 0.05). Across all phases of testing, most reaction characteristics, including cutaneous findings, gastrointestinal symptoms, cardiovascular involvement, neurologic manifestations, and route of drug administration, were not associated with depression, anxiety, stress, or DrHy-Q scores, either pre- or post-ST, placebo, or DPT procedures.

In multivariable analyses adjusted for sex, age group, and number of prior HSRs, age group was independently associated with DPT-post stress scores, with patients aged 40–59 years exhibiting significantly higher stress levels compared with those aged ≥60 years (*p* = 0.043). No independent associations were observed for depression or anxiety. In contrast, the number of prior HSRs emerged as an independent predictor of DPT-post DrHy-Q scores, indicating greater impairment in drug hypersensitivity-related QoL with increasing reaction history (*p* < 0.001). These results are summarized in Table 5.

In additional linear mixed-effects analyses, with assessment time, sex, age group, and number of prior HSRs included as fixed effects and a subject-specific random intercept, the overall effect of assessment time was significant for depression (F(5, 495) = 14.048, *p* < 0.001), anxiety (F(5, 495) = 14.584, *p* < 0.001), stress (F(5, 495) = 15.667, *p* < 0.001), and DrHy-Q (F(5, 495) = 27.134, *p* < 0.001). Age group was significantly associated with depression (F(2, 95) = 3.968, *p* = 0.022) and stress (F(2, 95) = 4.752, *p* = 0.011). DrHy-Q was significantly associated with sex (F(1, 95) = 7.091, *p* = 0.009) and the number of prior HSRs (F(1, 95) = 8.658, *p* = 0.004), whereas age group was not significantly associated with DrHy-Q (*p* = 0.817). No significant effects of sex, age group, or number of prior HSRs were observed for anxiety (all *p* > 0.05). Detailed mixed-effects model results are presented in Supplementary Table S3.

Before ST, patients who later avoided the drug despite negative DPT results (n = 16) had significantly higher baseline depression and anxiety scores than those who did not avoid drug use (n = 84) (*p* = 0.040 and *p* = 0.022, respectively), as well as higher DrHy-Q scores indicating worse drug hypersensitivity-related QoL (*p* = 0.014), while baseline stress scores were comparable between groups (*p* = 0.075). After DPT, patients who avoided the drug exhibited significantly higher depression, anxiety, stress, and DrHy-Q scores compared with patients who did not avoid drug use (*p* < 0.001).

## 4. Discussion

This study demonstrates that the diagnostic work-up for suspected IDHRs is associated with a consistent reduction in psychological burden. Depression, anxiety, stress, and DrHy-Q impairment decreased after each diagnostic phase (ST, placebo, and DPT), indicating that the evaluation process itself may contribute to psychological relief. Notably, improvements were observed not only post-DPT but also following placebo administration, suggesting that reassurance, structured monitoring, and perceived safety play an important role in alleviating anticipatory distress, even in the absence of active drug exposure, consistent with evidence from placebo and nocebo research highlighting the influence of expectation and clinician–patient interaction on emotional responses during medical interventions [26]. Importantly, direct, supervised exposure during in vivo testing may reduce uncertainty and fear by familiarizing patients with both the clinical environment and the diagnostic procedures. This experiential component, which cannot be replicated by in vitro assessments alone, may contribute to the progressive psychological improvement observed throughout the diagnostic work-up and underscores the value of in vivo tests beyond their confirmatory role.

Notably, psychological scores were already lower before DPT than before the preceding diagnostic stages, suggesting that the observed improvement may reflect a cumulative process throughout the diagnostic work-up rather than an effect specific to DPT. Increasing familiarity with the clinical setting, reassurance during the preceding diagnostic steps, and the passage of time may also have contributed to this progressive reduction. Thus, DPT should be interpreted as the final component of a sequential diagnostic process rather than as the sole determinant of the observed psychological improvement.

The largest reductions in psychological scores occurred post-DPT, suggesting that diagnostic clarification may contribute to psychological improvement. Prior studies have reported decreases in anxiety and depressive symptoms following DPT, highlighting the role of uncertainty and anticipatory fear in shaping psychological burden [19,27]. Confirmation of drug tolerance or the availability of a safe alternative may therefore reduce concerns related to future drug exposure. These findings support the established role of DPT as the diagnostic gold standard and suggest that, as part of the stepwise diagnostic process, it may also contribute to psychological improvement. Although several changes in psychological scores reached statistical significance, the magnitude of change was modest for some outcomes. Therefore, statistical significance should not necessarily be interpreted as clinical relevance, and the clinical relevance of these changes warrants further evaluation.

Reaction severity was not consistently associated with psychological burden across the diagnostic process. Higher anaphylaxis grades showed only weak and inconsistent associations with anxiety and stress, while no meaningful relationship was observed with DrHy-Q. These findings suggest that the psychological burden in suspected DHRs may be influenced by factors beyond the severity of prior reactions. In contrast, the cumulative number of previous reactions was consistently associated with significantly greater impairment in DrHy-Q scores. Similarly, Gastaminza et al. demonstrated that patients with suspected drug allergies who had experienced multiple reactions had worse drug hypersensitivity-related QoL, while Macy et al. showed that repeated drug reactions and multiple drug intolerances are associated with increased psychological burden and concerns about medication use [5,28]. Nevertheless, Görgülü Akın et al. demonstrated that drug hypersensitivity-related QoL significantly improved after the first three drug desensitization procedures, indicating that repeated clinical interventions may help alleviate the QoL burden associated with drug hypersensitivity [29].

Age-related differences were observed for stress, with patients aged 40–59 years exhibiting higher stress levels across diagnostic stages. Previous studies have shown that midlife is characterized by increased exposure to cumulative psychosocial stressors, which may influence stress responses independently of depressive or anxiety symptoms [21,30]. No consistent age-related differences were observed for depression, anxiety, or the DrHy-Q.

Most clinical characteristics, including time interval from the most recent HSRs, symptom onset time, clinical manifestations, route of drug administration, and atopy, were not associated with psychological outcomes. These negative findings align with broader evidence showing that objective symptom features are weak predictors of emotional burden in somatic conditions and that psychological responses are influenced more strongly by cognitive–affective processes such as uncertainty, expectation, and prior experience [26,31,32]. Psychological burden and impaired QoL have also been reported in other clinical conditions, including dermatovenerological diseases [33].

Female patients exhibited higher anxiety and DrHy-Q impairment, particularly during the early stages of the diagnostic evaluation. This finding is in line with previous reports showing that females tend to report higher levels of anxiety and health-related quality-of-life impairment in chronic allergic conditions, including asthma and DHR, although the underlying mechanisms may differ across diseases [34,35]. In the present study, sex-related differences in anxiety diminished post-DPT, suggesting that structured diagnostic clarification may reduce anticipatory distress. However, persistently higher DrHy-Q scores indicate that drug-related quality-of-life impairment may not fully normalize despite diagnostic evaluation, consistent with studies demonstrating that quality-of-life limitations can persist even after objective reassurance or successful desensitization [29].

Multivariable analyses demonstrated that post-DPT psychological outcomes were influenced by distinct patient-related factors. While post-DPT depression and anxiety scores were not independently associated with sex, age group, or prior reaction history, stress remained significantly associated with age group, with higher scores observed in middle-aged patients. Age-related differences in emotional responses to medical stressors have been previously reported and may reflect variations in psychosocial burden and coping strategies across the lifespan [21,30]. In contrast, drug hypersensitivity-related QoL followed a different pattern, as the number of prior HSRs emerged as the strongest independent predictor of post-DPT DrHy-Q scores. This finding is consistent with earlier studies showing that repeated adverse drug experiences were associated with persistent impairment in the DrHy-Q, even after diagnostic evaluation and reassurance [29]. Together, these results suggested that psychological distress and drug hypersensitivity-related QoL represented related but distinct domains, driven by different underlying determinants in the context of drug allergy assessment.

In the present study, an important finding was that patients who continued to avoid the provoked drug despite negative DPT results exhibited higher baseline and persistent post-DPT psychological burden, suggesting that diagnostic reassurance alone may be insufficient for a subset of patients. This observation highlights the need for targeted attention to individuals with higher baseline depression and anxiety symptoms and poorer DrHy-Q, who may benefit from additional supportive strategies integrated into drug allergy work-ups, such as structured education, brief counseling, or referral for psychological support when appropriate. In selected cases, a follow-up visit and, if clinically justified, repeat supervised drug exposure may further reinforce confidence in using medications confirmed as safe during DPT.

Although the current study provides valuable clinical insights, several limitations should be acknowledged. This was a single-center study, which may limit the generalizability of the findings to other clinical settings. The study included only adults with suspected IDHRs referred for specialist evaluation, while patients with non-immediate reactions and those meeting the predefined exclusion criteria were not included. In addition, the predominance of women and patients with severe reactions should be considered when generalizing these findings to other populations with suspected IDHRs. The relatively modest sample size and absence of an a priori sample-size calculation may have limited the ability to detect small effects and the robustness of subgroup analyses. Although the multivariable models were restricted to a limited number of clinically relevant covariates, the sample size may still affect model stability; therefore, these analyses should be considered exploratory. Bonferroni adjustment was applied to post hoc pairwise comparisons following the Friedman tests; nevertheless, the exploratory nature of the secondary analyses should be considered when interpreting these findings. Psychological outcomes were assessed using self-administered questionnaires rather than structured psychiatric interviews; therefore, these measures reflect psychological distress rather than clinical psychiatric diagnoses. Although the exclusion of patients with significant psychiatric comorbidity was intended to reduce confounding from pre-existing psychiatric conditions, it may have resulted in a population with a lower psychological burden and may limit the generalizability of the findings to patients with psychiatric comorbidity. Given the limited sample size, the potential influence of additional confounding factors could not be fully explored. Longer-term studies are needed to determine whether the observed psychological improvement is sustained over time. In addition, the observational design and absence of a control group preclude causal inference regarding the psychological effects of the diagnostic work-up. Therefore, the observed reductions in psychological scores cannot be attributed solely to the diagnostic procedures and may, at least in part, reflect regression to the mean, repeated questionnaire administration, reassurance, or the passage of time. Nevertheless, the longitudinal, stepwise assessment across skin testing, placebo administration, and drug provocation testing provides a detailed temporal evaluation of psychological changes during the diagnostic process.

## 5. Conclusions

This study demonstrates that the stepwise in vivo diagnostic evaluation for suspected IDHRs is associated with a consistent reduction in psychological burden and drug-related quality-of-life impairment. Depression, anxiety, stress, and DrHy-Q scores decreased progressively across ST, placebo administration, and DPT, with the greatest reduction in scores observed post-DPT.

These findings highlight that in vivo diagnostic procedures may provide benefits beyond diagnostic clarification by contributing to psychological reassurance. Patients with multiple prior reactions may represent a subgroup requiring additional psychological support during the diagnostic process. Incorporating awareness of psychological burden into routine drug allergy assessment may enhance the overall effectiveness of the diagnostic process.

## Figures and Tables

**Figure 1:**
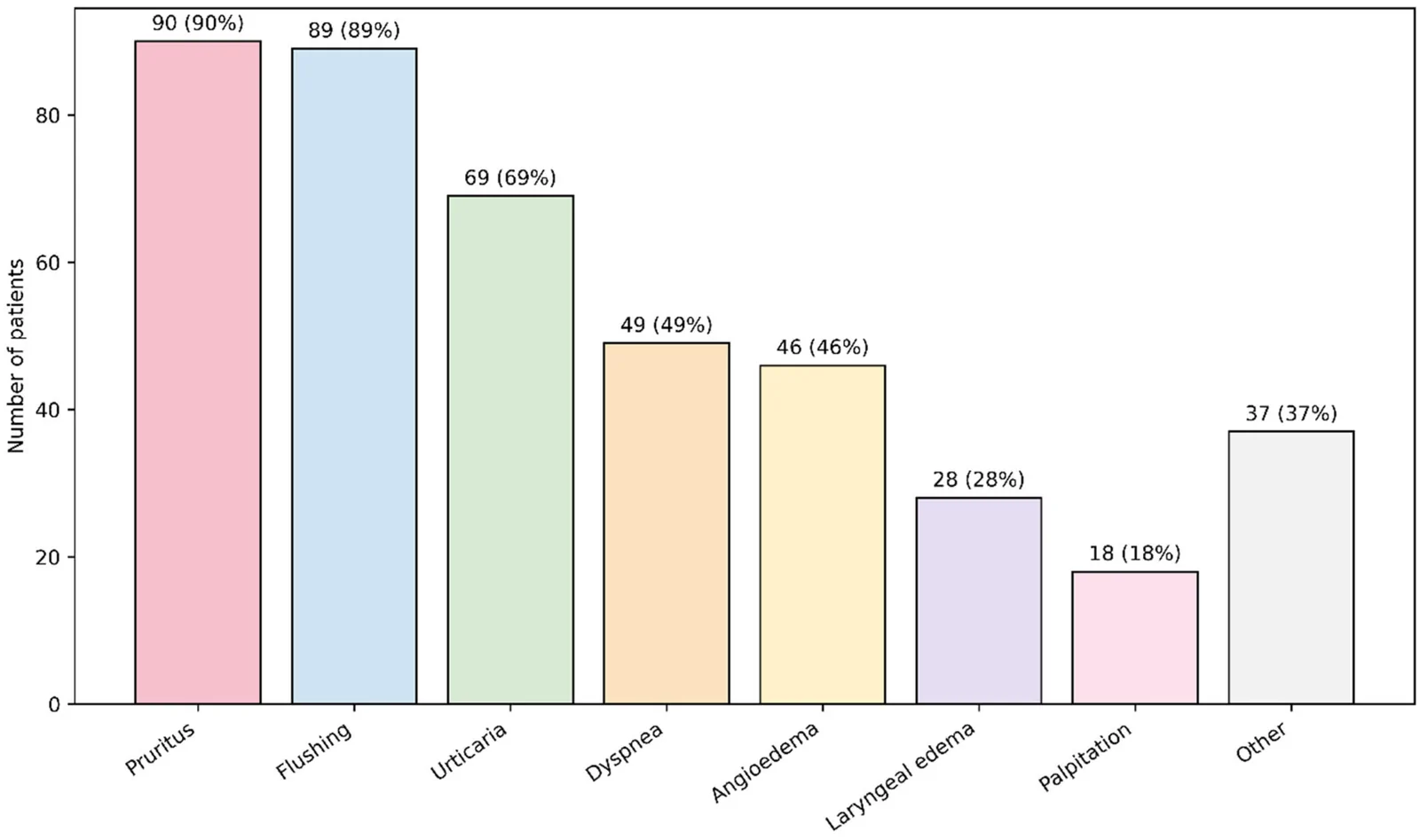
Distribution of clinical manifestations among patients with a history of immediate hypersensitivity reactions. Other includes hypotension (9%), neurologic symptoms (12%), emesis (10%), vomiting (4%), chest pain (1%), and cardiopulmonary arrest (1%).

**Figure 2:**
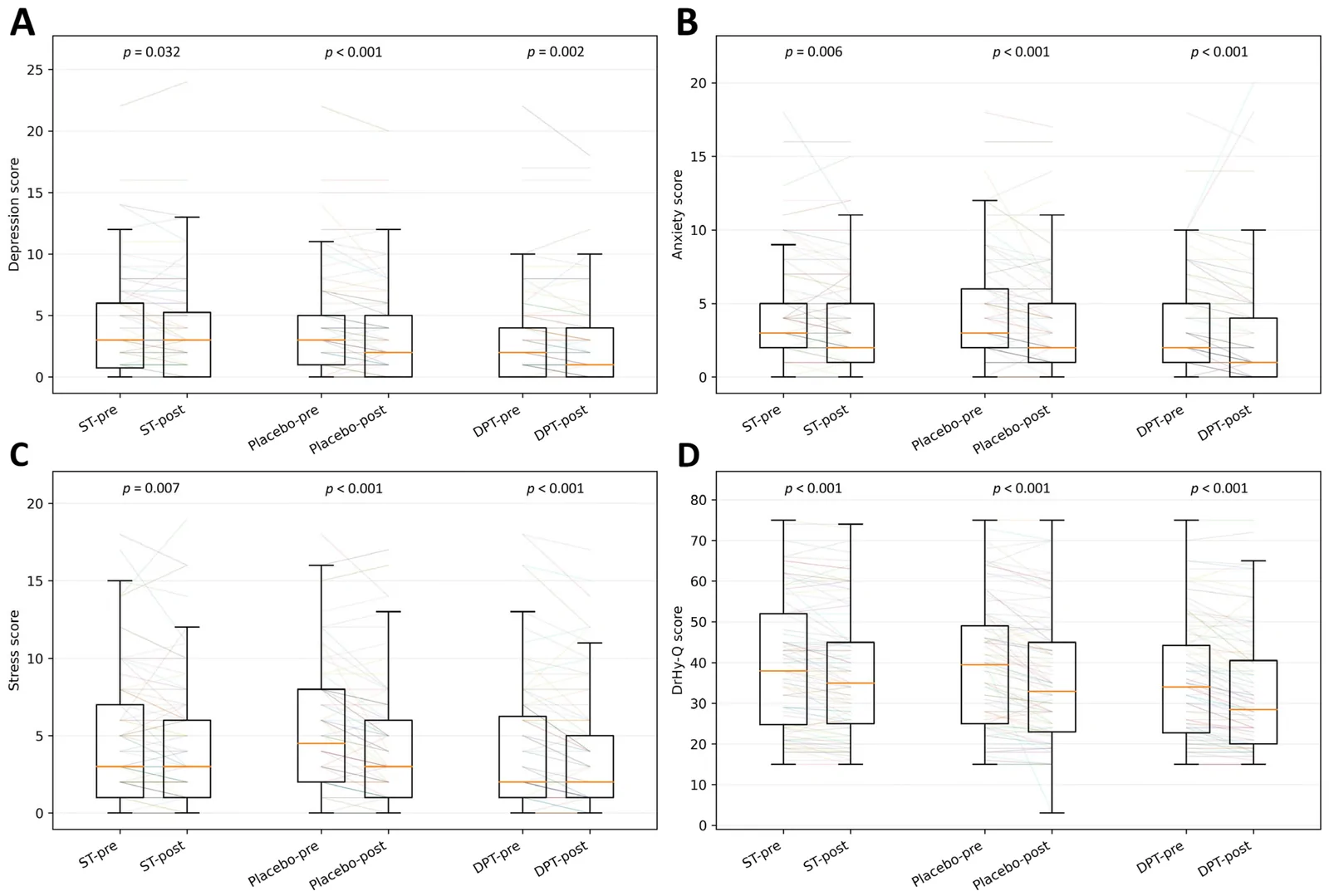
Changes in psychological and DrHy-Q scores during the diagnostic work-up: (**A**) Depression, (**B**) anxiety, (**C**) stress (DASS-21 subscales), and (**D**) DrHy-Q scores measured before and after ST, placebo administration, and DPT. Box plots display medians and interquartile ranges; thin lines represent individual patient trajectories. *p* values indicate within-phase pre–post comparisons. Abbreviations: DPT, drug provocation test; DrHy-Q, Drug Hypersensitivity Quality of Life Questionnaire; ST, skin test.

**Figure 3:**
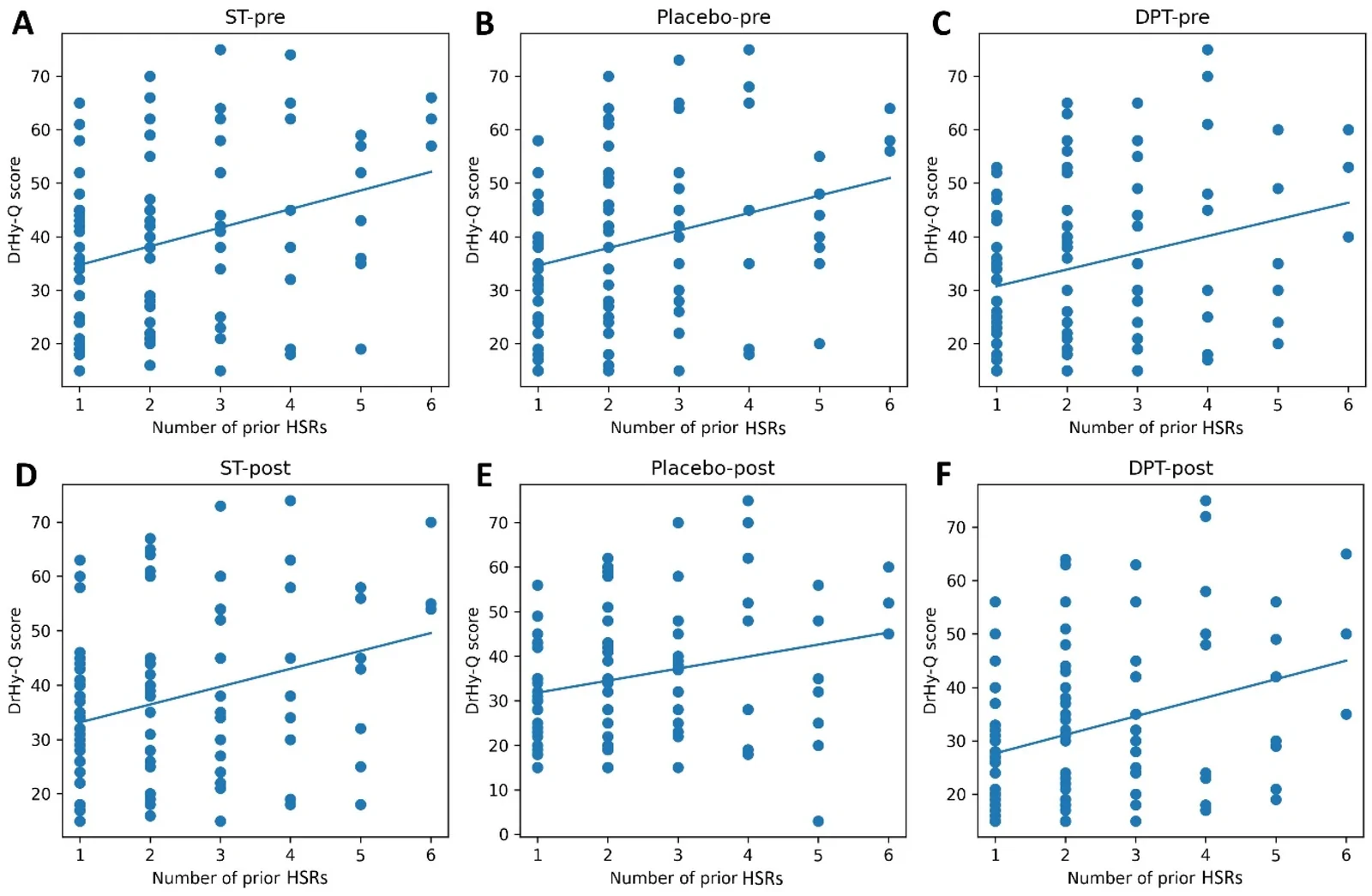
Correlation between the number of prior HSRs and DrHy-Q scores across diagnostic work-up. Panels (**A**–**C**) show pre-test assessments before ST, placebo administration, and DPT, respectively, while panels (**D**–**F**) show post-test assessments after the corresponding procedures. Significant positive correlations were observed between prior HSR count and DrHy-Q scores across all diagnostic stages (ST-pre: ρ = 0.269, *p* = 0.007; ST-post: ρ = 0.249, *p* = 0.012; placebo-pre: ρ = 0.290, *p* = 0.003; placebo-post: ρ = 0.245, *p* = 0.014; DPT-pre: ρ = 0.290, *p* = 0.003; DPT-post: ρ = 0.328, *p* = 0.001). Each panel displays individual patient data with a fitted trend line illustrating the association between reaction count and DrHy-Q scores. Abbreviations: DPT, drug provocation test; DrHy-Q, Drug Hypersensitivity Quality of Life Questionnaire; HSRs, hypersensitivity reactions; ST, skin test.

**Figure 4:**
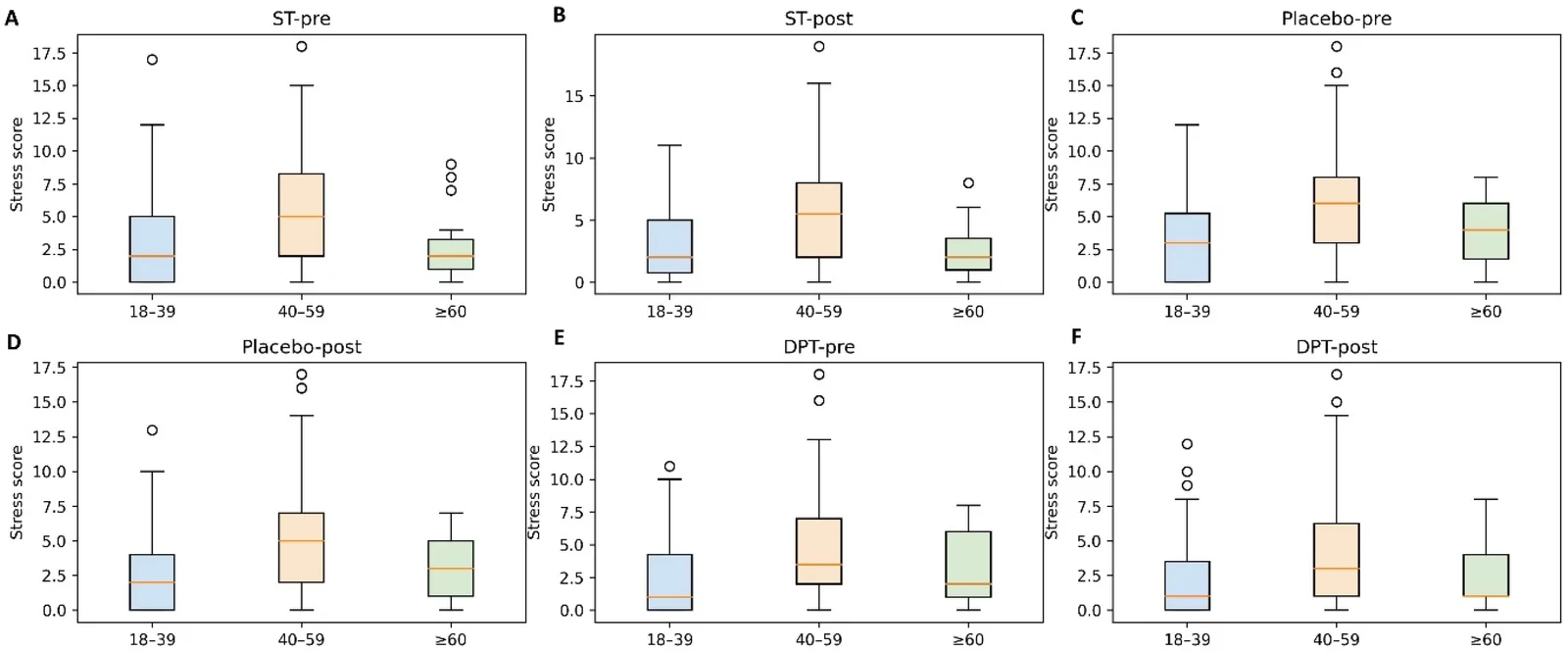
Comparison of stress scores among age groups across diagnostic work-up. Panels (**A**–**F**) show stress scores across age groups at different stages of the diagnostic work-up: ST-pre (**A**), ST-post (**B**), placebo-pre (**C**), placebo-post (**D**), DPT-pre (**E**), and DPT-post (**F**). Box plots show median and interquartile ranges of stress scores across age groups at each diagnostic stage. Abbreviations: DPT, drug provocation test; ST, skin test.

**Table 1:** Clinical and demographic characteristics of the patients.

Parameters	Results
Age (years), mean ± SD	44.2 ± 12.5
**Sex, *n* (%)**	
Female	72 (72)
Male	28 (28)
**Presence of atopy, *n* (%)**	22 (22)
House dust mite	14 (14)
Other	8 (8)
**Occupation, *n* (%)**	
Homemaker	46 (46)
White-collar workers	28 (28)
Blue-collar workers	10 (10)
Retired	9 (9)
Students	5 (5)
Healthcare workers	2 (2)
**Comorbidities, *n* (%)**	44 (44)
Asthma	9 (9)
Hypertension	16 (16)
Diabetes mellitus	12 (12)
Hypothyroidism	9 (9)
Other *	11 (11)

* Chronic gastritis, Hashimoto’s thyroiditis, migraine, fibromyalgia, and ischemic heart disease. Boldface and gray shading are used to distinguish main categorical variables from their corresponding subcategories and do not indicate statistical significance.

**Table 2:** Clinical features of the reported hypersensitivity reactions.

Parameters	Results
History of anaphylaxis**, *n* (%)**	51 (51)
**Adrenaline administration, *n* (%)**	
Administered	8 (8)
Not administered	85 (85)
Unknown	7 (7)
**Severity of HSRs, *n* (%)**	
Grade 1	7 (7)
Grade 2	42 (42)
Grade 3	46 (46)
Grade 4	3 (3)
Grade 5	2 (2)
**Time interval from the index HSRs, months, median (IQR)**	24 (6.8–60)
≤6 months**, *n* (%)**	25 (25)
6–12 months**, *n* (%)**	17 (17)
>12 months**, *n* (%)**	58 (58)
**Time interval from the most recent HSRs, months, median (IQR)**	6 (3–12)
≤6 months**, *n* (%)**	51 (51)
6–12 months**, *n* (%)**	27 (27)
>12 months**, *n* (%)**	22 (22)
**Number of Prior HSRs, median (IQR)**	2 ( 1–3)
1 reaction**, *n* (%)**	35 (35)
2 reactions**, *n* (%)**	29 (29)
≥3 reactions**, *n* (%)**	36 (36)
**Symptom onset, *n* (%)**	
0–1 h	59 (59)
1–6 h	41 (41)
**Drug administration route, *n* (%)**	
Oral	85 (85)
Intravenous	18 (18)
Intramuscular	3 (3)
Subcutaneous	1 (1)
Topical	4 (4)
**Suspected culprit drugs, *n* (%)**	
β-lactams antibiotics	44 (44)
NSAIDs	42 (42)
Non-β-lactam antibiotics	20 (20)
Multivitamin preparation	9 (9)
PPIs	6 (6)
Other drugs	15 (15)

Abbreviations: HSRs, hypersensitivity reactions; IQR, interquartile range; NSAIDs, nonsteroidal anti-inflammatory drugs; PPIs, proton pump inhibitors. Boldface and gray shading are used to distinguish main categorical variables from their corresponding subcategories and do not indicate statistical significance.

**Table 3:** Comparison of psychological measures before and after clinical interventions.

Measure	Phase	Pre-Test	Post-Test	*p*-Value
**Depression**				
	STs	3.0 (0.25–6.0)	3 (0.0–5.75)	0.032
	Placebo	3 (1.0–5.0)	2 (0.0–5)	<0.001
	DPT	2 (1.0–4.0)	1 (0.0–4)	0.002
**Anxiety**				
	STs	3.0 (2.0–5.0)	2 (1.0–5.0)	0.006
	Placebo	3(2.0–6.0)	2 (1.0–5.0)	<0.001
	DPT	2 (1.0–5.0)	1 (0.0–4)	<0.001
**Stress**				
	STs	3 (1.0–7.0)	3 (1.0–6.0)	0.007
	Placebo	4.5 (2.0–8.0)	3 (1.0–6.0)	<0.001
	DPT	2 (1.0–6.75)	2 (1.0–5)	<0.001
**DrHy-Q**				
	STs	38 (24–52)	35 (25–45)	<0.001
	Placebo	39.5 (25–49)	33 (23–45)	<0.001
	DPT	34 (22.25–44.75)	28.5 (20–41)	<0.001

Continuous variables are presented as median (25th–75th percentiles). *p* values refer to within-phase comparisons. Abbreviations: DPT, drug provocation tests; DrHy-Q, Drug Hypersensitivity Quality of Life Questionnaire; STs, skin tests. Boldface and gray shading indicate the main outcome measures and do not denote statistical significance.

**Table 4:** Comparison of psychological outcomes and DrHy-Q across diagnostic stages.

Outcome	Phase	Friedman Test	Significant Differences * (Bonferroni-Adjusted)	No Significant Difference *
**Depression**	Pre-test	*p* < 0.001	DPT < ST (*p* < 0.001) DPT < Placebo (*p* < 0.001)	ST vs. Placebo (*p* = 1.000)
	Post-test	*p* < 0.001	DPT < ST (*p* < 0.001) DPT < Placebo (*p* = 0.001)	ST vs. Placebo (*p* = 1.000)
**Anxiety**	Pre-test	*p* < 0.001	DPT < ST (*p* < 0.001) DPT < Placebo (*p* < 0.001)	ST vs. Placebo (*p* = 1.000)
	Post-test	*p* < 0.001	DPT < ST (*p* < 0.001) DPT < Placebo (*p* < 0.001)	ST vs. Placebo (*p* = 0.437)
**Stress**	Pre-test	*p* < 0.001	DPT < ST (*p* = 0.003) DPT < Placebo (*p* < 0.001)	ST vs. Placebo (*p* = 0.102)
	Post-test	*p* < 0.001	DPT < ST (*p* < 0.001) DPT < Placebo (*p* < 0.001)	ST vs. Placebo (*p* = 0.697)
**DrHy-Q**	Pre-test	*p* < 0.001	DPT < ST (*p* < 0.001) DPT < Placebo (*p* < 0.001)	ST vs. Placebo (*p* = 0.442)
	Post-test	*p* < 0.001	DPT < ST (*p* < 0.001) DPT < Placebo (*p* < 0.001)	ST vs. Placebo (*p* = 0.155)

* In the “Significant differences” column, “A < B” indicates that scores in phase A were significantly lower than in phase B. “A vs. B” indicates no significant difference between phases A and B. Abbreviations: DPT, drug provocation tests; DrHy-Q, Drug Hypersensitivity Quality of Life Questionnaire; STs, skin tests. Boldface and gray shading are used to distinguish the main outcome measures and table headings and do not indicate statistical significance.

**Table 5:** Multivariable linear regression analyses of post-DPT psychological outcomes.

Outcome	Independent Predictor	Coefficient (β)	95% CI	*p*-Value
**Depression**				
	Age (40–59)	2.12	−0.27 to 4.51	0.082
	Age (18–39)	−0.24	−2.68 to 2.20	0.846
	Female	0.45	−1.35 to 2.25	0.622
	Number of Prior HSRs	0.07	−0.54 to 0.67	0.829
**Anxiety**				
	Age (40–59)	2.17	−0.19 to 4.53	0.071
	Age (18–39)	0.71	−1.70 to 3.12	0.561
	Female	0.04	−1.73 to 1.82	0.964
	Number of Prior HSRs	0.10	−0.50 to 0.70	0.748
**Stress**				
	Age (40–59)	1.97	0.25 to 3.65	0.043
	Age (18–39)	−0.01	−2.32 to 2.31	0.996
	Female	0.11	−1.59 to 1.82	0.895
	Number of Prior HSRs	0.33	−0.25 to 0.91	0.258
**DrHy-Q**				
	Age (40–59)	−3.64	−11.78 to 4.50	0.377
	Age (18–39)	−1.98	−10.29 to 6.34	0.639
	Female	5.88	−0.25 to 12.02	0.060
	Number of Prior HSRs	3.43	1.36 to 5.50	<0.001

Multivariable linear regression models were adjusted for sex, age group, and number of prior HSRs. The ≥60 year age group and male sex were used as reference categories. Abbreviations: DPT, drug provocation tests; DrHy-Q, Drug Hypersensitivity Quality of Life Questionnaire; HSRs, hypersensitivity reactions. Boldface and gray shading are used to distinguish the main outcome measures and table headings and do not indicate statistical significance.

## Data Availability

The data that support the findings of this study are available from the corresponding author upon reasonable request. The data are not publicly available due to privacy and ethical restrictions.

## Supplementary Materials

The following supporting information can be downloaded at: https://www.mdpi.com/article/10.3390/jcm15207964/s1, Table S1: Associations between anaphylaxis severity, age groups, and psychological measures across diagnostic stages; Table S2: Sex-based comparison of psychological scores across test phases; Table S3: Linear mixed-effects model analyses of psychological outcomes and DrHy-Q scores across the diagnostic process.

## Abbreviations

The following abbreviations are used in this manuscript:

ADRs	Adverse drug reactions
DASS-21	Depression Anxiety Stress Scale-21
DHRs	Drug hypersensitivity reactions
DPT	Drug provocation test
DrHy-Q	Drug Hypersensitivity Quality of Life Questionnaire
HSRs	Hypersensitivity reactions
IDHRs	Immediate drug hypersensitivity reactions
MDAS	Multiple drug allergy syndrome
MDIS	Multiple drug intolerance syndrome
NIHRs	Non-immediate hypersensitivity reactions
NSAIDs	Nonsteroidal anti-inflammatory drugs
QoL	Quality of life
ST	Skin test
WAO	World Allergy Organization
