# Supplementary material for: Changes in Psychological Burden and Quality of Life During In Vivo Diagnostic Evaluation for Immediate Drug Hypersensitivity: A Prospective Stepwise Study
Source: J Clin Med. 2026 Oct 15;15(20):7964. doi: 10.3390/jcm15207964 (PMC13706027; doi:10.3390/jcm15207964)
Supplement: Supplementary file 1 [file jcm-15-07964-s001.zip › jcm-4552813-supplementary.pdf]

Supplementary Table S1. Associations between anaphylaxis severity, age groups, and psychological measures across diagnostic stages.

Outcome	Phase	Prior HSRs (ρ)	Prior HSRs (p-value)	HSR grade (ρ)	HSR grade (p-value)	Age groups (p-value)
**Depression**						
	ST-pre	0.081	0.422	-0.163	0.105	0.106
	ST-post	0.111	0.270	-0.150	0.136	0.095
	Placebo-pre	0.042	0.678	-0.159	0.114	0.244
	Placebo-post	0.066	0.513	-0.167	0.096	0.249
	DPT-pre	0.049	0.630	-0.168	0.095	0.068
	DPT-post	0.068	0.504	-0.156	0.122	0.098
**Anxiety**						
	ST-pre	0.067	0.511	-0.236	0.018	0.510
	ST-post	0.128	0.203	-0.195	0.052	0.368
	Placebo-pre	0.114	0.260	-0.236	0.018	0.279
	Placebo-post	0.157	0.119	-0.184	0.067	0.214
	DPT-pre	0.122	0.227	-0.134	0.184	0.428
	DPT-post	0.780	0.439	-0.094	0.350	0.309
**Stress**						
	ST-pre	0.158	0.115	-0.232	0.021	0.012
	ST-post	0.171	0.089	-0.254	0.011	0.013
	Placebo-pre	0.157	0.119	-0.224	0.025	0.023
	Placebo-post	0.192	0.056	-0.221	0.027	0.008
	DPT-pre	0.105	0.297	-0.253	0.011	0.046
	DPT-post	0.157	0.119	-0.204	0.042	0.029
**DrHyQ**						
	ST-pre	0.269	0.007	-0.109	0.281	0.756
	ST-post	0.249	0.012	-0.132	0.189	0.827
	Placebo-pre	0.290	0.003	-0.098	0.331	0.979
	Placebo-post	0.245	0.014	-0.075	0.460	0.975

DPT-pre	0.290	0.003	-0.101	0.316	0.726
DPT-post	0.328	0.001	-0.073	0.469	0.941

DPT; Drug provocation tests, DrHy-Q; Drug Hypersensitivity Quality of Life Questionnaire, HSRs; Hypersensitivity reactions, STs; Skin tests. Boldface and gray shading indicate the main outcome measures and do not denote statistical significance.

Supplementary Table S2. Sex-based comparison of psychological scores across test phases

Outcome	Phase	Female Pre-Test	Male Pre-Test	p-value	Female Post-Test	Male Post-Test	p-value
**Depression**							
	STs	3.0 (1.0 – 6.0)	1.0 (0.0 – 4.5)	0,042	3.0 (1.0 – 6.0)	1.0 (0.0 – 4.0)	0,054
	Placebo	3.0 (1.0 – 6.75)	1.0 (0.0 – 3.75)	0,031	3.0 (1.0 – 5.75)	1.0 (0.0 – 3.75)	0,022
	DPT	2.0 (0.0 – 5.0)	1.0 (0.0 – 3.0)	0,149	1.5 (0.0 – 4.75)	1.0 (0.0 – 3.0)	0,308
**Anxiety**							
	STs	3.0 (2.0 – 6.0)	2.0 (0.0 – 4.0)	0,020	3.0 (2.0 – 6.0)	2.0 (0.0 – 3.0)	0,010
	Placebo	3.0 (2.0 – 6.0)	2.0 (0.0 – 4.75)	0,005	2.5 (1.0 – 6.0)	2.0 (0.0 – 3.75)	0,019
	DPT	2.0 (1.0 – 5.0)	1.5 (0.0 – 3.75)	0,152	1.0 (0.25 – 5.0)	1.0 (0.0 – 3.5)	0,326
**Stress**							
	STs	3.5 (2.0 – 8.0)	2.0 (0.25 – 5.75)	0,054	3.0 (1.25 – 7.0)	1.0 (0.25 – 5.75)	0,055
	Placebo	5.0 (2.0 – 8.0)	2.5 (0.0 – 6.75)	0,069	3.0 (2.0 – 6.75)	2.0 (0.0 – 5.0)	0,065
	DPT	2.0 (1.0 – 6.0)	1.5 (0.0 – 7.09)	0,280	2.0 (1.0 – 5.0)	1.5 (0.0 – 5.5)	0,393
**DrHy-Q**							
	STs	42.0 (27.2 – 57.0)	30.5 (22.5 – 42.2)	0,012	38.5 (26.5 – 55.7)	28.0 (20.0 – 39.5)	0,003
	Placebo	42.0 (28.5 – 54.2)	29.5 (22.0 – 40.2)	0,002	37.5 (25.7 – 48.0)	26.0 (20.0 – 34.0)	0,003
	DPT	35.0	24.0	0,026	30.5	26.0	0,024

(25.2 – 47.2)	(19.0 – 39.7)		(24.0 – 43.7)	(20.0 – 34.0)

P values refer to within-phase comparisons. DPT; Drug provocation tests, DrHy-Q; Drug Hypersensitivity Quality of Life Questionnaire, STs; Skin tests. Boldface and gray shading indicate the main outcome measures and do not denote statistical significance.

Supplementary Table S3. Linear mixed-effects model analyses of psychological outcomes and DrHy-Q scores across the diagnostic process.

Outcome	Fixed effect	F	df	p-value
**Depression**				
	Assessment time	14.048	5, 495	<0.001
	Sex	0.631	1, 95	0.429
	Age group	3.968	2, 95	0.022
	Number of prior HSRs	0.021	1, 95	0.884
**Anxiety**				
	Assessment time	14.584	5, 495	<0.001
	Sex	2.369	1, 95	0.127
	Age group	1.768	2, 95	0.176
	Number of prior HSRs	0.263	1, 95	0.610
**Stress**				
	Assessment time	15.667	5, 495	<0.001
	Sex	1.157	1, 95	0.285
	Age group	4.752	2, 95	0.011
	Number of prior HSRs	0.862	1, 95	0.356
**DrHy-Q**				
	Assessment time	27.134	5, 495	<0.001
	Sex	7.091	1, 95	0.009
	Age group	0.202	2, 95	0.817
	Number of prior HSRs	8.658	1, 95	0.004

Note: Linear mixed-effects models were fitted separately for each outcome. Assessment time comprised six repeated measurements (ST-pre, ST-post, placebo-pre, placebo-post, DPT-pre, and DPT-post) and was included as a fixed effect together with sex, age group, and number of prior hypersensitivity reactions (HSRs). A subject-specific random intercept was included to account for within-participant correlation. Models were estimated using restricted maximum likelihood with the Satterthwaite approximation for degrees of freedom. Bold values

indicate statistical significance ($p < 0.05$). Boldface and gray shading indicate the main outcome measures and do not denote statistical significance.
